# *De novo* transcriptome assembly reveals sex-specific selection acting on evolving neo-sex chromosomes in *Drosophila miranda*

**DOI:** 10.1186/1471-2164-15-241

**Published:** 2014-03-27

**Authors:** Vera B Kaiser, Doris Bachtrog

**Affiliations:** 1 Department of Integrative Biology, Center for Theoretical Evolutionary Genomics, University of California, Berkeley, Berkeley, CA 94720, USA; 2 Current Address: MRC Human Genetics Unit, MRC Institute of Genetics and Molecular Medicine, University of Edinburgh, Western General Hospital, Edinburgh EH4 2XU, UK

**Keywords:** Sex chromosomes, Drosophila, Transcriptome, Gene loss, Nonsense mediated decay

## Abstract

**Background:**

The *Drosophila miranda* neo-sex chromosome system is a useful resource for studying recently evolved sex chromosomes. However, the neo-Y genomic assembly is fragmented due to the accumulation of repetitive sequence. Furthermore, the separate assembly of the neo-X and neo-Y chromosomes into genomic scaffolds has proven to be difficult, due to their low level of sequence divergence, which in coding regions is about 1.5%. Here, we *de novo* assemble the transcriptome of *D. miranda* using RNA-seq data from several male and female tissues, and develop a bioinformatic pipeline to separately reconstruct neo-X and neo-Y transcripts.

**Results:**

We obtain 2,141 transcripts from the neo-X and 1,863 from the neo-Y. Neo-Y transcripts are generally shorter than their homologous neo-X transcripts (N50 of 2,048-bp vs. 2,775-bp) and expressed at lower levels. We find that 24% of expressed neo-Y transcripts harbor nonsense mutation within their open reading frames, yet most non-functional neo-Y genes are expressed throughout all of their length. We find evidence of gene loss of male-specific genes on the neo-X chromosome, and transcriptional silencing of testis-specific genes from the neo-X.

**Conclusions:**

Nonsense mediated decay (NMD) has been implicated to degrade transcripts containing pre-mature termination codons (PTC) in Drosophila, but rampant description of neo-Y genes with pre-mature stop codons suggests that it does not play a major role in down-regulating transcripts from the neo-Y. Loss or transcriptional down-regulation of genes from the neo-X with male-biased function provides evidence for beginning demasculinization of the neo-X. Thus, evolving sex chromosomes can rapidly shift their gene content or patterns of gene expression in response to their sex-biased transmission, supporting the idea that sex-specific or sexually antagonistic selection plays a major role in the evolution of heteromorphic sex chromosomes.

## Background

The differentiation of an evolving pair of sex chromosomes involves many evolutionary steps, including changes in gene content on the X and Y [1,2]; gene loss and the fixation of deleterious mutations on the Y [3]; epigenetic modifications related to silencing of Y-linked genes [4] and the evolution of dosage compensation [5,6]. The *Drosophila miranda* neo-sex chromosome system is emerging as a useful resource to study the evolution of young sex chromosomes [7-9]. *D. miranda* split from its sister species *D. pseudoobscura* only about 2 my (million years) ago, and has sex chromosomes of different ages: Muller A (referred to as XL), the ancestral sex chromosome shared among all Drosophila species including *D. melanogaster*, is at least 60 my old [10,11]; Muller D (referred to as XR), a neo-sex chromosome system of intermediate age (about 15 my) and shared with *D. pseudoobscura*; Muller C (referred to as neo-X/neo-Y; about 1–2 my old), specific to the *D. miranda* lineage.

The genome sequence of *D. miranda* has recently been published, including a draft assembly of the neo-Y chromosome [9]. A major challenge in the assembly of this chromosome was the fact that the neo-Y has already accumulated many repetitive sequences, which are difficult to assemble – similar to the ancestral Y chromosome of Drosophila which has not been assembled at all. In addition, the high level of sequence identity between the neo-X and neo-Y especially in coding regions further complicates assemblies using next-generation sequencing data, since many short sequencing reads are identical from the neo-X and neo-Y. To annotate the neo-Y and study expression changes, diagnostic SNPs between the neo-X and neo-Y were used to estimate the relative abundance of neo-sex linked transcripts [9]. This, however, does not allow studying the structure of the neo-Y transcripts *per se*. In particular, it is unknown how complete neo-Y protein-coding genes are, especially with regards to the transcripts that contain premature stop codons or frameshift mutations. Furthermore, the use of diagnostic SNPs for expression analysis will lead to an inherent mapping bias towards higher neo-X expression, since RNA-Seq reads can overlap with parts of transcripts that differ structurally between the neo-X and neo-Y. Using the neo-X sequence as a baseline for comparison, one can only study neo-Y transcripts that are also present on the neo-X and show no major structural changes or indels. Thus, there is considerable interest to study the neo-sex transcriptome directly, which we do here.

In addition to providing insights into sex chromosome evolution, the neo-sex chromosomes of *D. miranda* also allow us to study other cellular phenomena, such as Nonsense-mediated mRNA decay (NMD). NMD is a cellular surveillance mechanism that degrades transcripts containing premature termination codons (PTCs) [12-14]. Destruction of transcripts containing PTCs by NMD prevents production of truncated, potentially harmful proteins that may interfere with normal cellular processes. The exact mechanism through which NMD recognized PTCs in Drosophila is unclear, though the length of the 3′ UTR has been implicated [12], whereas the presence of an exon-exon junction downstream of a stop codon is an initiator of NMD in mammals [15]. Given that a large number of neo-Y transcripts contain PTCs, the *D. miranda* system makes an excellent case for testing if the length of the 3′UTR leads to NMD in flies.

A major challenge in constructing the neo-sex transcriptome is that coding regions on the neo-X and neo-Y are very similar; based on the genome sequence, the estimated divergence at coding regions is only about 1.5%. Accordingly, using Illumina RNA-Seq reads for transcriptome assembly, not all reads can be assigned to the neo-X or neo-Y unambiguously. This problem is for the most part irrelevant for the neo-X transcriptome, which can be assembled directly in females (but note that this approach will miss male-specific neo-X transcripts). In males, however, transcriptome assemblers fail to assemble the neo-X and neo-Y into separate transcripts. Instead, chimeric transcripts are produced that contain partial neo-X and partial neo-Y sequence, connected by regions that are not differentiated between the neo-sex chromosomes. To obtain an assembly of the neo-Y transcriptome, we developed a bioinformatic pipeline, making use of genomic read mapping against the neo-sex transcripts in males.

## Methods

### Assembly pipeline

We used Illumina Genome Analyzer II paired-end 100-bp RNA-seq reads of the inbred line MSH22 to assemble the *D. miranda* transcriptome with Trinity [16]. First, low-quality sections of RNA-Seq reads and remaining adaptor sequence were discarded, and the minimum contig size of the Trinity assembly was set to 200 bp. For the neo-Y assembly, 157 million RNA-Seq reads from four different samples (virgin male whole body, virgin male gonadectomized carcass, virgin testis and virgin accessory gland; NCBI accession numbers SRR364803, SRR364802, SRR364798, SRR364799) were combined in a single assembly; similarly, to obtain neo-X transcripts, 163 million RNA-Seq reads from virgin ovary, virgin female whole body and virgin female gonadectomized carcass were combined and assembled together (NCBI accession numbers SRR364804, SRR364801, SRR364800). For the autosomes and old sex chromosomes (XL and XR), both male and female reads were combined for a single assembly. The *D. pseudoobscura* transcriptome (strain MV25) was assembled *de novo* using combined virgin male and virgin female whole body samples (87 million reads that had been randomly sampled from the Illumina Hiseq 2000 paired-end reads with NCBI accession numbers SRR357403 and SRR357405).

**Figure 1:**
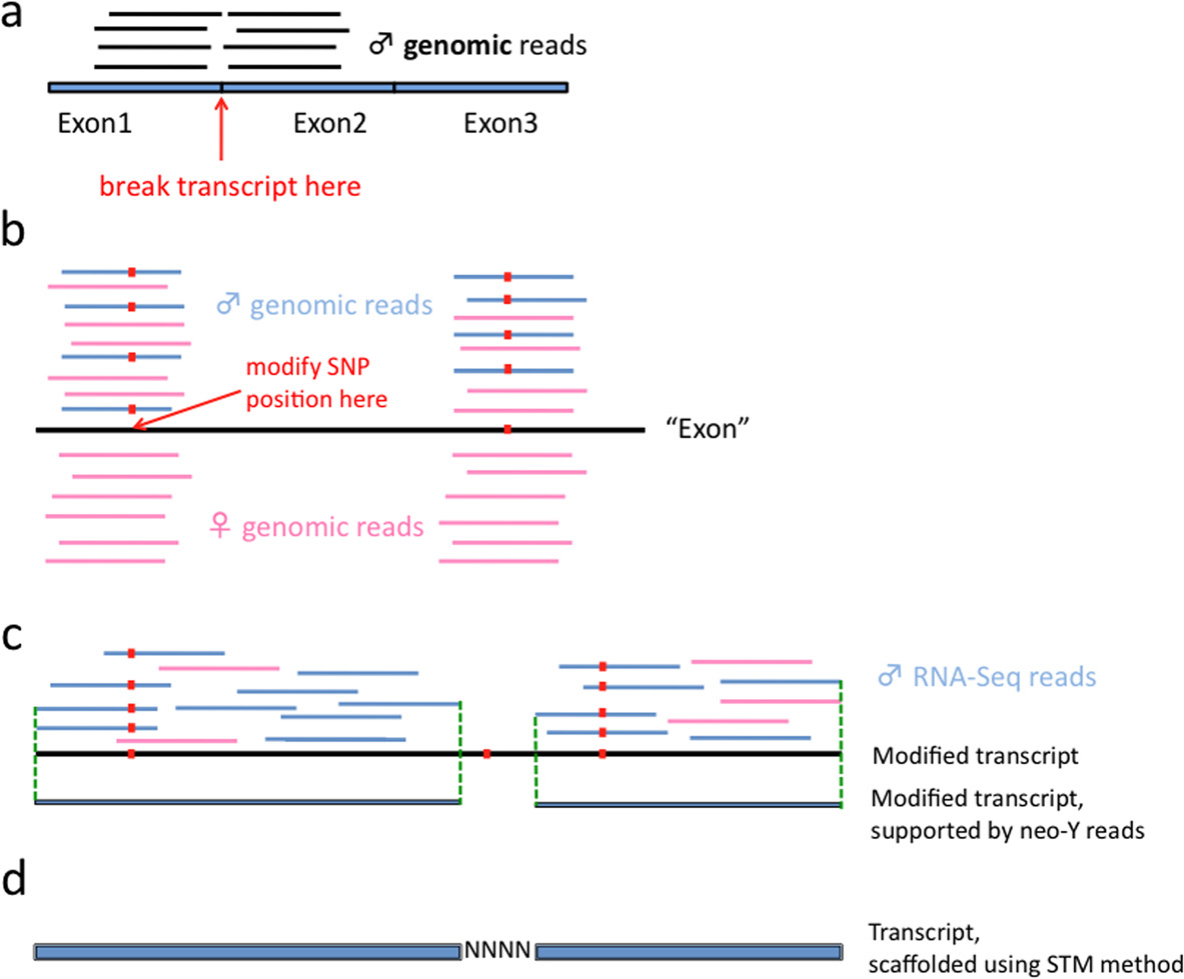
**Neo-Y assembly pipeline. (a)** Trinity *de novo* transcripts were broken into exons using genomic read mapping information; **(b)** transcripts were modified to only harbor neo-Y specific variants; **(c)** only sections of neo-Y transcripts were kept if they were supported by RNA-Seq reads and carried at least one variant compared to the neo-X; **(d)** transcripts were scaffolded using the STM method and *D. pseudoobscura* protein alignments.

An overview of the pipeline to assemble the neo-Y transcripts is given in Figure 1. Using blastn, Trinity transcripts in males were compared to the *D. miranda* genome assembly [9]. Potential Muller C transcripts (blastn score of > 200) were kept and modified to become neo-Y transcripts, by introducing variants that distinguish the neo-X from the neo-Y.

For this means, male genomic reads were compared to the potential Muller C transcripts and SNP positions were identified. Note that, in contrast to RNA-Seq reads, male genomic reads contain equal proportions of neo-X and neo-Y variants in males. To avoid introducing erroneous variants at intron-exon junctions, male Trinity transcripts were first divided up into exons. This was done by mapping male genomic reads against the male Muller C transcripts using Mosaik (http://code.google.com/p/mosaik-aligner/), allowing for zero mismatches between the reads and transcripts. Any parts of the transcripts for which mapped reads overlapped by less than 6 bp were considered hypothetical exon-exon junctions, and transcripts were split into “exons” at these positions. Using *D. pseudoobscura* gene models, it was confirmed that exon-exon junctions were correctly identified in a random test sample of transcripts. Next, both male and female genomic reads were mapped against the exons of the male Muller C transcripts using Mosaik, this time allowing for mismatches between the genomic reads and transcripts (in Mosaik, the penalty for gap opening, gap extension and the mismatch score were set to 10, 6.6 and −5 respectively). Freebayes (arXiv:1207.3907) was used to call SNPs in the mapping of male and female genomic reads versus Muller C exons (median coverage of 45 and 62 respectively). SNPs were called when 3 or more reads for the alternative alleles with a base quality of >13 were present at a given site, or if the coverage was 9 or less, the minimum number of reads supporting the alternative alleles was set to two.

Based on the freebayes output, SNPs and indels detected by mapping genomic reads against the exons of transcripts were categorized into the following three classes (category 1 and category 3 SNPs are divergent sites between the neo-X and neo-Y, while category 2 SNPs are caused by either mapping or sequencing errors, or remaining heterozygosity in the inbreed lines used for sequencing):

1. At a given position, male reads were polymorphic, and all female reads showed a variant that was different compared to the reference “exon” sequence (0.25% of all sites). These sites were assumed to already contain the neo-Y variant in the transcript sequence.

2. At a given position, both male and female reads showed a SNP or indel; this could be caused by polymorphisms on the neo-X, sequencing error or by reads from paralogous locations being wrongly mapped to that particular position in both sexes; these positions were left unchanged (0.21% of all sites).

3. At a given position, male reads were polymorphic, whereas female reads showed no SNP or indel (1.1% of all sites). Such a site was considered to contain the neo-X variant in the transcript sequence and was modified to contain the neo-Y variant, using a perl script.

Genomic neo-Y BAC sequences [17] were used to heuristically fine-tune the Mosaik mapping parameters as well as the freebayes SNP calling parameters, so as to minimize the neo-Y versus BAC sequence divergence. Our pipeline makes the implicit assumption that neo-X genomic reads can be mapped back to neo-Y transcripts, which allows the identification of neo-X/neo-Y diverged sites. This assumption may be violated in regions where divergence is high enough for neo-X reads not to map, even if the homologous sequence is still present on the neo-X chromosome. However, such regions are unlikely to affect the neo-Y transcripts in the final assembly, since for regions of high divergence, Trinity will assemble neo-X and neo-Y transcripts separately. Our pipeline only modifies parts of a transcript where the neo-X and neo-Y are similar enough for neo-X reads to align to the neo-Y, and highly diverged regions will not be modified by our bioinformatics pipeline.

To test how this bioinformatic pipeline performs, we also mapped male and female reads against autosomal transcripts, and categorized variants in the same manner as for the neo-sex linked genes. We find that 0.03% of autosomal sites would fall into category 1, and 0.15% in category 3, i.e. the proportions of such sites relative to those for neo-sex-linked transcripts are 12% and 13%, respectively. This suggests that about 12-13% of the sites on Muller element C that appear as “male polymorphic and female homomorphic” may be caused by factors other than true neo-X/neo-Y divergence. We find that 0.24% of autosomal sites would fall into category 2 (compared to 0.21% for the neo-sex linked transcripts). Since the proportions of category 2 SNPs are very similar between neo-sex chromosomes and autosomes, this is consistent with SNPs in this category being caused by mapping and sequencing errors, or remaining heterozygosity in the line, rather than true neo-X/neo-Y divergence.

After modification of the male Muller C transcripts, the male RNA-Seq reads were re-mapped against the modified transcripts, allowing for zero mismatches between reads and transcripts. Based on this mapping and the freebayes SNP/Indel output, transcript regions were kept for further analysis if they were supported by male RNA-Seq reads (i.e. actually present in the male transcriptome) and if they contained at least one variant distinguishing the neo-Y from the neo-X. Requiring at least one neo-Y-specific variant per contiguous transcript region has the consequence that genes that were deleted from the neo-Y were removed from the neo-Y assembly, as were neo-X/neo-Y homomorphic genes and silenced neo-Y genes.

Using this approach alone, we missed male-specific neo-X transcripts because they were turned into neo-Y transcripts; however, male-specific neo-X transcripts were assembled in an analogous way to the neo-Y transcripts (Figure 1), except that this time positions were modified to carry the variant that was also found in females (the neo-X variant).

All transcripts were merged using Cap3 [18], and then scaffolded based on *Drosophila pseudoobscura* known proteins using the STM method [19]. All transcripts were assigned to Flybase polypeptide IDs, using a reciprocal best hit between the assembled transcripts and *D. pseudoobscura* Flybase transcripts as a criterion for ID assignment.

### Transcript level calculation

To calculate transcript abundance for the neo-X and neo-Y separately, RNA-Seq reads from male carcass, whole body, testis and accessory gland were aligned to the *D. miranda* transcriptome assembly using Mosaik and allowing for zero mismatches between the reads and the reference transcripts. eXpress [20] probabilistically assigns reads to different alleles of the same gene – in this case, the neo-X and neo-Y homologs – and was used to calculate transcript abundance levels (measured as FPKM - the fragments per kilobase of transcript per million fragments mapped) for the neo-sex chromosomes separately, as well as for genes on other Muller elements.

### Sequence analysis of transcripts

Neo-X and neo-Y genes, as well as their autosomal *D. pseudoobscura* homologs were aligned using muscle [21]. Gblocks [22] was used to remove poorly aligned segments of alignments, and the minimum length of a block was set to 100, and the maximum number of consecutive non-conserved positions to four. Next, only alignments that contained all three sequences (neo-X, neo-Y and *D. pseudoobscura*) were considered (1,646 alignments). For these transcripts, distmat (http://emboss.bioinformatics.nl/cgi-bin/emboss/distmat) was used to calculate the uncorrected divergence between the neo-X, neo-Y and *D. pseudoobscura.*

To assess the functional properties of neo-Y genes, the neo-Y transcripts were aligned to the protein sequences of their *D. pseudoobscura* homologs using Genewise [23]. For the alignments, genewise allows for frame-shift mutations and pre-mature stop codons in the transcript sequences, and a perl program was used to extract the positions of these mutations within the neo-Y transcripts.

For each neo-Y transcript, the length of the 3′ UTR was calculated by the distance between the first stop codon to the end of the transcript. Since neo-Y expression is generally low, we also used the length of the 3′UTR of the neo-X homolog as a proxy for UTR length on the neo-Y, assuming that the neo-Y transcript might not be fully assembled if the read number supporting that part of the transcript was too low.

### Availability of supporting data

The data sets supporting the results of this article are available in the ncbi Sequence Read Archive (SRA) repository, http://www.ncbi.nlm.nih.gov/sra/?term=SRR364803, http://www.ncbi.nlm.nih.gov/sra/?term=SRR364802, http://www.ncbi.nlm.nih.gov/sra/?term=SRR364798, http://www.ncbi.nlm.nih.gov/sra/?term=SRR364799, http://www.ncbi.nlm.nih.gov/sra/?term=SRR364804, http://www.ncbi.nlm.nih.gov/sra/?term=SRR364801, http://www.ncbi.nlm.nih.gov/sra/?term=SRR364800, http://www.ncbi.nlm.nih.gov/sra/?term=SRR357403, http://www.ncbi.nlm.nih.gov/sra/?term= SRR357405.

## Results and discussion

### The neo-sex transcriptome

The raw Trinity assembly of the male transcriptome contained 67,872 transcripts, 17,220 of which showed sequence similarity (blat Score >200) to Muller element C of the *D. miranda* genomic assembly [9]. It was manually confirmed that neo-sex transcripts in males were produced as chimeric transcripts by Trinity, by comparing several transcripts to neo-Y and neo-X BAC clone sequences of ref. [17]. The female Trinity assembly contained 43,730 transcripts, 7,046 of which potentially neo-X linked. 1,568 of the male Muller C matching transcripts had exact matches with the neo-X assembly, suggesting that Trinity assembled some neo-X transcripts correctly, in addition to chimeric and correctly assembled neo-Y transcripts. The 17,220 potential Muller C transcripts from males were divided into 31,368 exonic sequences. By mapping of genomic reads followed by SNP calling, about 1.1% of all sites in Muller C transcripts from males were transformed into the corresponding neo-Y variant.

**Figure 2:**
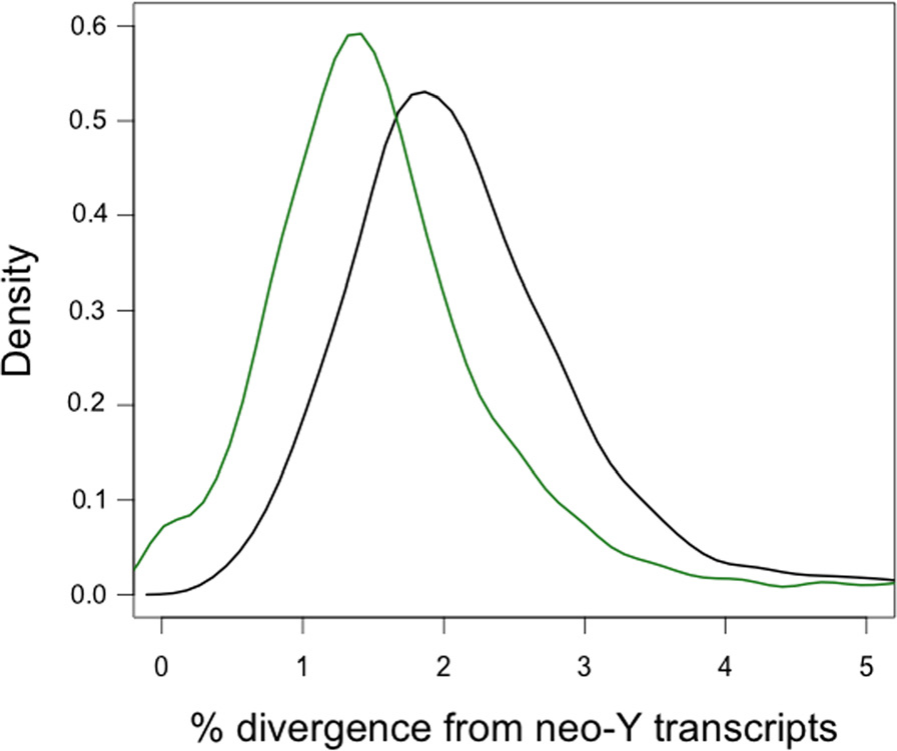
**Density plot of the total divergence between the assembled neo-Y transcripts compared to neo-X (green) and *D. pseudoobscura *** **(black) transcripts, respectively.**

All transcripts were scaffolded using the STM method and *D. pseudoobscura* protein sequences (flybase: dpse-all-translation-r2.26.fasta), which contains 2,851 annotated polypeptides on Muller C. The final *D. miranda* transcriptome assembly, which has been submitted to NCBI under the accession number GALP00000000, contained 12,522 protein-coding genes, including 2,141 from the neo-X, 1,863 from the neo-Y and 8,500 from the autosomes/ancestral sex chromosomes. Thus, our *de novo* transcriptome captures a large fraction of genes present on the neo-sex chromosomes, with 1,754 protein-coding genes being expressed from both the neo-X and neo-Y, 387 transcripts only being detected from the neo-X, and 109 transcripts only from the neo-Y. N50 is a statistical measure of the average length of a set of sequences, defined as the length *N* for which 50% of all bases in the sequences are in a sequence of length *L* < *N*; N50 was 2,775 bp for the neo-X; 2,048 bp for the neo-Y; 2,800 bp for the autosomes and the older X chromosomes combined. Thus, neo-Y transcripts were, on average, shorter compared to those from the neo-X or autosomes (Wilcoxon test comparing the lengths of neo-X *versus* neo-Y transcripts: W = 1657390, *p* < 10^−15^). Nevertheless, we obtain nearly as many transcripts from the neo-Y as from the neo-X. The median sequence divergence between the neo-Y and neo-X was 1.5% and between the neo-Y and *D. pseudoobscura* 2.1%, suggesting that the neo-sex chromosome system in *D. miranda* was formed shortly after the split of the two species, rather than at the time of speciation (Figure 2).

Using genewise, neo-Y transcripts were divided into those with putative functional ORFs and those that are non-functional. Of the 1,410 neo-Y transcripts with no detectable frame-shift mutations or premature stop codons, the median length was 1,249 – somewhat shorter than for transcripts annotated as containing disrupted ORFs by frameshift mutations (356 transcripts) or pre-mature stop codons (164 transcripts) – combined, 453 putative non-functional neo-Y genes, with a median length of 1,649 bp (Wilcoxon test comparing the lengths of putative functional *versus* non-functional neo-Y transcripts: W = 234222, *p* < 10^−15^). Genes containing nonsense or frameshift mutations might simply be longer, on average, because they present larger mutational targets, but notably, they are still transcribed throughout most of their length. On the other hand, some knock-out mutations might have been missed if they were not part of the assembled transcript sequence. Indeed, compared to the genome assembly [9], we detect fewer loss-of-function mutations among neo-Y-linked genes (Additional file 1); however, we also find mutations in 170 genes that were annotated as “functional” in the genomic assembly (Additional file 1). Partly, this may be due to the relatively high chance of missing a pseudogene in the genome assembly - which was estimated as 10% [9]. In addition, we followed a different strategy of assigning flybase polypeptide IDs to neo-Y transcripts, and the two assemblies may differ with regards to which exact transcript is assigned to which FBpp ID (in the genome assembly, co-linearity and scaffold information was taken into account, whereas the transcripts were assigned based on a reciprocal best hit with *D. pseudoobscura* genes only). However, since all loss-of function mutations in the transcriptome assembly are supported by genomic as well as RNA-Seq reads, they are presumably real.

### Nonsense mediated decay

**Figure 3:**
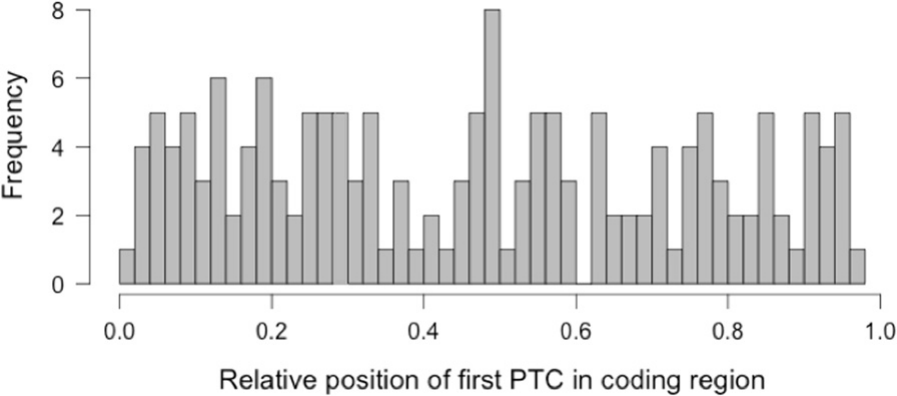
Histogram of the position of the first pre-terminal codon (PTC) within the neo-Y coding sequence, relative to the length of the neo-Y coding region.

The median length of the 3′UTR for the neo-X sequences was 346-bp, slightly shorter than the median UTR length of *D. pseudoobscura* transcripts (405-bp) [24]. In contrast, for neo-Y genes without PTCs, the 3′UTRs had a median length of only 112-bp, i.e. neo-Y 3'UTRs were substantially shorter compared to the neo-X 3′UTRs (Wilcoxon Test: W = 922411.5, *p* < 10^−5^). In part, this may be caused by Trinity not being able to fully assemble neo-Y UTRs that are lowly expressed. Among the 164 neo-Y genes with PTCs, median original UTR length was only 22.5-bp when counting from the ancestral stop codon to the end of the transcript, but it was increased to 666.5-bp when counting from the first PTC to the end of the transcript. Accordingly, stop codons often occurred in the beginning of the transcript sequence, and appear distributed randomly across the coding sequence of a gene (Figure 3). However, several lines of evidence suggest that NMD does not play a major role in down-regulating transcript levels of neo-Y genes that contain PTCs.

**Figure 4:**
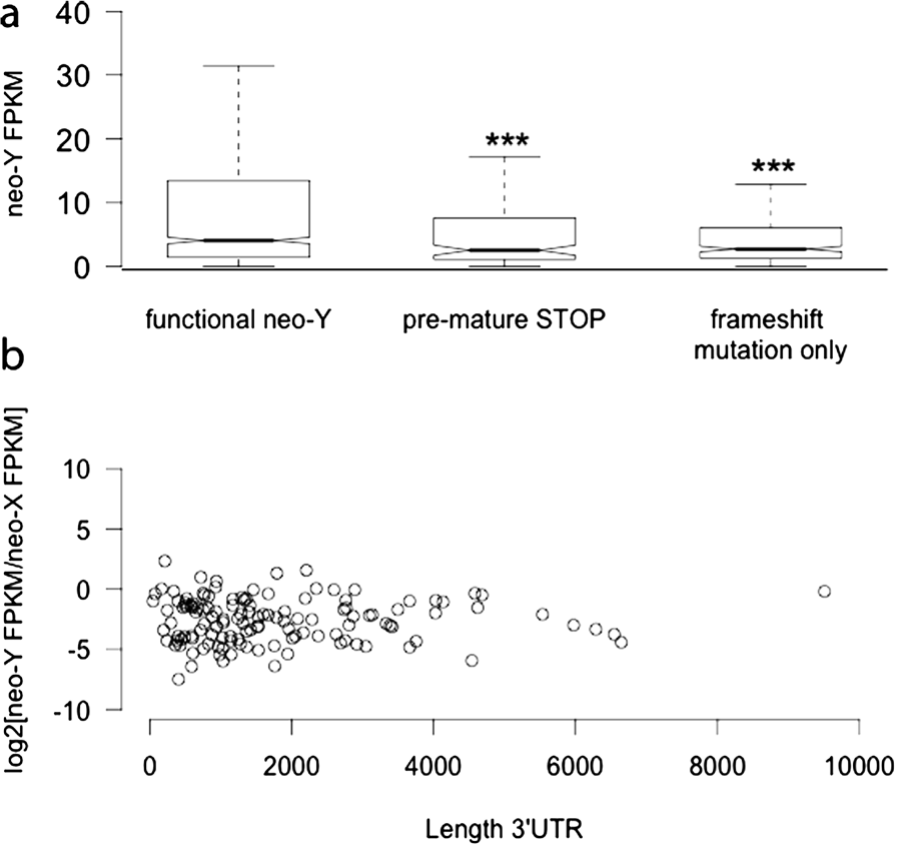
**No evidence for nonsense-mediated decay in reducing neo-Y transcript abundance. (a)** Neo-Y FPKM levels for transcripts with intact ORFs, transcripts containing a PTC and transcripts containing a frame-shift mutation only (and no PTC). Transcript levels for both classes of putative non-functional genes were significantly decreased, compared to those with intact ORFs (Wilcoxon test: W = 132628, *p* < 0.001 and W = 234562, *p* < 0.001). **(b)** Transcript abundance of the neo-Y in males, relative to the neo-X homolog in females, as a function of the length of the 3*'*UTR (estimated as the sum of the corresponding neo-X UTR length, plus the distance from the first PTC to the end of the CDS).

First, FPKM-values were reduced for non-functional neo-Y genes, irrespective of whether they contained a frame-shift mutation only without causing a PTC further down-stream of the transcript, or if they contained a PTC (Figure 4a). Accordingly, most down-regulation of non-functional genes occurs irrespective of NMD, which suggest general mechanisms such as epigenetic modifications causing reduced expression of non-functional genes [4], rather than down-regulation due to feedback in the cell after transcripts have been produced. In line with this, neo-Y genes that were not present in the *de novo* transcriptome assembly were often annotated as functional by ref. [9] (54% of annotated silenced genes), and only 15% contained PTCs, i.e. down-regulation by NMD cannot explain the lack of expression for the majority of these silenced transcripts. Furthermore, for transcripts containing PTCs, there was no detectable relationship between 3′UTR length and transcript levels (Figure 4b).

Thus, our results suggest that NMD does not play a major role in down-regulating neo-Y transcript levels in *D. miranda* if PTCs are present. This is consistent with possibly abundant read-through of stop codons in Drosophila, as indicated by protein-coding conservation 3′ of stop codons [25]. In order to infer if translation proceeds past the pre-mature stop codons of neo-Y-linked genes, direct measures of translation are necessary, such as ribosomal profiling. Interestingly, neo-X genes in *D. miranda* show significantly higher transcript levels if they contain UTR lengths larger than the median (female whole body FPKM = 11.24 for genes with 3′UTRs < 424.5-bp, compared to an FPKM = 23.5 for neo-X genes with 3'UTRs > 425-bp, Wilcoxon test: W = 470328, p < 10^−4^). If longer 3′UTRs are indeed associated with higher levels of expression (and not due to less complete assemblies of lowly expressed genes), this may counteract any effects of NMD.

### Gene loss and silencing on the neo-X chromosome

The availability of neo-Y transcripts allowed us to identify genes that were lost from the neo-X since its split from the neo-Y about 1.5 my ago. When mapping female genomic reads back to male transcripts, four Muller C transcripts had zero female genomic read coverage, indicating that these genes are not present in the female genome. There was also no polymorphism among male genomic reads mapped to these transcripts, further supporting the idea that only a single, neo-Y-linked transcript was present in the genome. A fifth gene (FBpp0276435/FBpp0276436) was identified as having an unusually high “divergence” from BAC clone neo-Y sequences of ref. [17]. This gene turned out to be present in two copies in the neo-Y genomic assembly and also in two copies on Muller C in *D. pseudoobscura*, but only in one copy on the neo-X (Additional file 2).

**Figure 5:**
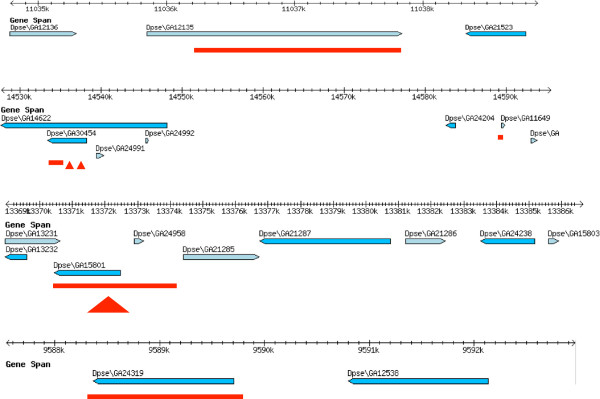
**Deletions on the neo-X chromosome in *D. miranda*.** Shown are flybase gbrowser plots of genomic regions on Muller element C in *D. pseudoobscura* (genome assembly version 3); red bars indicate deletions in the homologous regions on the neo-X in *D. miranda*, and red arrows indicate insertion on the neo-X. See Additional file 2 for a detailed description of the inferred deletions.

Whole genome alignments of *D. miranda*, *D. pseudoobscura* and *D. affinis*[26] were used to verify the deletions on the neo-X, and to investigate the scale of the deletion. In each case, neo-X deletions only affected single genes or parts of genes, suggesting that they were gene-specific and small in scale (Figure 5).

**Figure 6:**
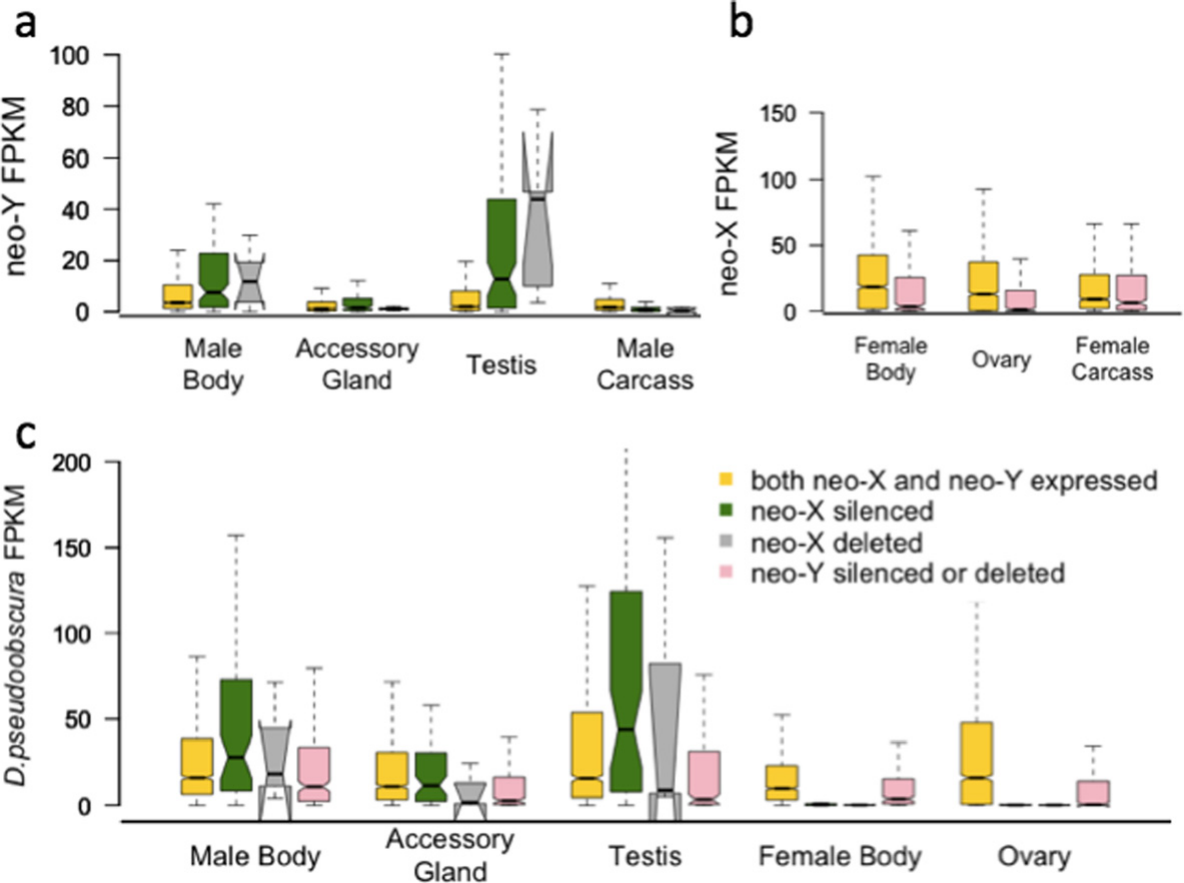
**Transcript abundance (FPKM) for different classes of neo-sex-linked genes. a)** Neo-Y FPKM-levels in *D. miranda* males; in testis, neo-Y transcripts whose homologues on the neo-X have been deleted or silenced are expressed at significantly higher levels compared to transcripts whose neo-X homologues are expressed (Wilcoxon test: W = 63263, *p* < 10^−8^ for neo-X deleted genes; W = 42141, *p* < 0.01 for neo-X silenced genes). **b)** Neo-X FPKM-levels in *D. miranda* females. **c)** FPKM-levels of *D. pseudoobscura* transcripts which are homologues to neo-sex linked *D. miranda* genes; in testis, *D. pseudoobscura* transcripts whose homologues on the neo-X in *D. miranda* have been transcriptionally silenced are expressed at significantly higher levels compared to transcripts whose homologues on the neo-X are expressed (W = 42141, *p* < 0.01); the same observation holds true when the three *D. pseudoobscura* transcripts with neo-X deleted homologues are added to a combined “neo-X silenced or deleted” category (W = 44476, *p* < 0.01).

Interestingly, the neo-Y homologs of genes that were lost on the neo-X chromosome are highly expressed from the testis in *D. miranda* (Figure 6a). Similarly, the 109 transcripts that were recovered from the neo-Y and not the neo-X show testis-biased expression in *D. miranda* males (Figure 6a). Expression levels of Muller C genes in *D. pseudoobscura* can be used as a proxy to infer their ancestral function, before becoming sex-linked in *D. miranda*. In the *D. pseudoobscura* transcriptome, homologs of only three of the five neo-X deleted genes could be retrieved; these, however, show almost no expression in females and moderate to high expression in male tissues (Figure 6c), suggesting that they were ancestrally male-specific in function. Likewise, genes that have been silenced on the neo-X in the *D. miranda* lineage (neo-X genes that are not part of the transcriptome but present in the genome) are also male-biased in expression in *D. pseudoobscura* (Figure 6c)*.* Demasculinization (that is, a deficiency of genes with male-biased expression) has been found on the ancestral X chromosome of Drosophila, and on chromosome XR of the *D. pseudoobscura* lineage [27]. Our observations of both beginning down-regulation of male-specific transcripts as well as targeted gene loss of male-specific genes from the young neo-X of *D. miranda* demonstrate that these changes can occur rapidly within a short evolutionary time frame.

In contrast, the 387 genes that were recovered from the neo-X but not the neo-Y (either because of neo-Y deletion or gene silencing) had generally low expression in *D. miranda* females and in both sexes of *D. pseudoobscura* (Figure 6b and c), supporting the idea that lowly expressed genes are lost at an increased rate from the neo-Y [2]. Mapping of male genomic reads against neo-X transcripts suggests that most silent genes on the neo-Y are indeed still present in the genome, with only their transcription being suppressed, i.e. their genomic coverage in males is only partially reduced (Additional file 3). Indeed only 30 of the 387 genes with silent neo-Y but expressed neo-X copies were annotated as deleted from the neo-Y [9].

## Conclusions

Evolved sex chromosomes have a highly diverged gene content [1,5,27]. Ancient Y chromosomes have usually lost most of their ancestral genes, and the few remaining genes often have testis-specific functions [1,5]. In contrast, old X chromosomes in Drosophila have become demasculinized, containing a deficiency of genes with male-specific expression [27]. The evolutionary events leading to such a difference in gene content, and their temporal dynamics, are little understood. Here, we show that both gene loss and gene silencing contribute to divergence in gene content between evolving sex chromosomes. Genes with male-specific function are preferentially silenced and lost on an evolving neo-X chromosome. On the other hand, lowly expressed genes are lost quickly from the neo-Y, while male-specific genes are more likely to be retained on the degenerating neo-Y chromosome. Thus, masculinization of the Y and demasculinization of the X can proceed very quickly after a new sex chromosome emerges, supporting the idea that sex-specific or sexually antagonistic selection plays a major role in the evolution of heteromorphic sex chromosomes [1,5,28].

## Competing interests

The authors declare that they have no competing interests.

## Authors’ contributions

VBK built the bioinformatic pipeline, performed the analyses and wrote the paper; DB conceived the study and contributed to the analysis and writing of the paper. Both authors read and approved the final manuscript.

## Supplementary Material

Additional file 1 **Venn diagram of the number of genes annotated as non-functional, i.e. containing frame-shift mutations or PTCs, in the *de novo *transcriptome assembly and the genome assembly of**[9]. This diagram is based on a total of 1,460 neo-Y genes that were allocated to the Muller C element in both assemblies, expressed from the neo-Y (i.e. present in the transcriptome) and had flybase polypeptide IDs assigned to them (i.e. “maker genes” of the genome assembly were excluded). Click here for file

Additional file 2 Detailed description of the neo-X deleted gene regions. Click here for file

Additional file 3 Genomic coverage of neo-X transcripts in males, for neo-X genes whose neo-Y homologs are not transcribed and possibly deleted (left) or present in the neo-Y transcriptome (right). Click here for file
