# Supplementary figures and images for: De novo transcriptome assembly reveals sex-specific selection acting on evolving neo-sex chromosomes in Drosophila miranda
Source: BMC Genomics. 2014 Mar 27;15:241. doi: 10.1186/1471-2164-15-241 (PMC3986819; doi:10.1186/1471-2164-15-241)

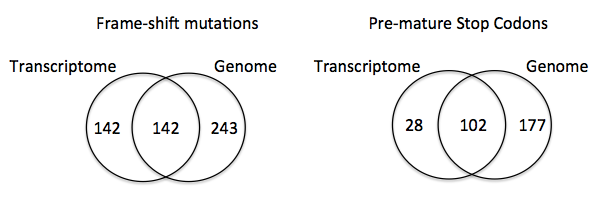

Supplement: Additional file 1 — Venn diagram of the number of genes annotated as non-functional, i.e. containing frame-shift mutations or PTCs, in the de novo transcriptome assembly and the genome assembly of[9]. This diagram is based on a total of 1,460 neo-Y genes that were allocated to the Muller C element in both assemblies, expressed from the neo-Y (i.e. present in the transcriptome) and had flybase polypeptide IDs assigned to them (i.e. “maker genes” of the genome assembly were excluded). [file 1471-2164-15-241-S1.tiff]

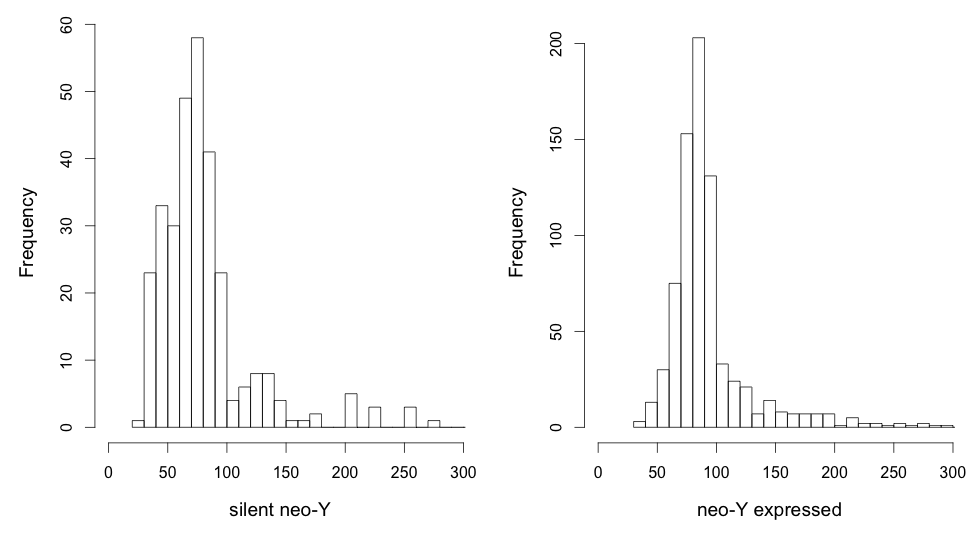

Supplement: Additional file 3 — Genomic coverage of neo-X transcripts in males, for neo-X genes whose neo-Y homologs are not transcribed and possibly deleted (left) or present in the neo-Y transcriptome (right). [file 1471-2164-15-241-S3.tiff]
