# Supplementary material for: De novo transcriptome assembly reveals sex-specific selection acting on evolving neo-sex chromosomes in Drosophila miranda
Source: BMC Genomics. 2014 Mar 27;15:241. doi: 10.1186/1471-2164-15-241 (PMC3986819; doi:10.1186/1471-2164-15-241)
Supplement: Additional file 2 — Detailed description of the neo-X deleted gene regions. [file 1471-2164-15-241-S2.docx]

Supplementary Information

Gene deletions on the neo-X chromosome:

The transcript of FBpp0278149 (GA12135) in *D. pseudoobscura* contains seven exons, located at 3:11035845..11037834; in *D. miranda,* the region spanning exon 2 to exon 7 is deleted but present in *D. pseudoobscura* and *D. affinis*. The neo-Y transcript is fully assembled in *D. miranda* males, including all seven exons, an ORF of 1,638bp (the same length as in *D. pseudoobscura*), and 5’ and 3’ UTR’s, suggesting that the neo-Y copy is fully functional.

FBpp0307340 (GA30454) (located at Muller 3:14533376..14538192) contains two exons in *D. pseudoobscura*. Whole genome alignments suggest that the neo-X copy in *D. miranda* has undergone one intragenic duplication of 84 bp within the first exon, and one 204 bp insertion of a nearby gene, FBpp0278441 (GA24991), within exon 2. The 404 bp region that is transcribed from the neo-Y chromosome in *D. miranda* is seemingly functional (no nonsense or frameshift mutations, compared to the *D. pseudoobscura* ORF), and it lies within a 750bp region of exon 2 that is deleted on the neo-X.

FBpp0278443(GA11649) is a single-exon transcript of 426bp, located at 3:14589398..14589823 in *D. pseudoobscura*, and the whole ORF is also present in *D. affinis*. On the *D. miranda* neo-X, the first 313 bases of the ORF are deleted, including 30bp upstream of the start codon.

In *D. pseudoobscura*, the transcripts FBpp0276186 and FBpp0276188 belong to the paralogous genes GA24238 and GA15801 respectively (OrthoDB6_Drosophila: EOG6MW8S9), with FBpp0276186 being located at 3:13383522..13385184 and FBpp0276188 nearby at 3:13370436..13372472. In *D. pseudoobscura*, the uncorrected divergence between the two transcripts is only 0.83%, suggesting that the duplication event occurred after the split with *D. miranda*. Indeed, whole genome alignments of the genomic region show that both *D. miranda* and *D. affinis* lack a copy of FBpp0276186. Further, FBpp0276188 is also completely deleted on the *D. miranda* neo-X, including 3kb upstream of the gene until the middle of its 3’ UTR. Instead of FBpp0276188, the *D. miranda* neo-X carries a 3kb insertion not found in the other two species, which contains a 400 bp DNA transposon sequence as identified by repeatmasker.org. The transcript expressed from the neo-Y is assumed to be homologous to FBpp0276188 (because this gene is also present in *D. affinis*), covers 425 bp, including parts of exon 2 and 3, and is potentially functional despite being lowly expressed (FPKM in male body = 3.9).

FBpp0276436/ FBpp0276435 are two adjacent paralogs on Muller C in *D. pseudoobscura* (3:9590796..9592133 for FBpp0276435/GA12538 and 3:9,588,367..9,589,704 for FBpp0276436/GA24319). The divergence between the two paralogs is 8.15%, suggesting that they existed before the *D. pseudoobscura -D. miranda* split. In whole genome alignments, FBpp0276435 is fully present on the neo-X; FBpp0276436, on the other hand, is completely absent on the neo-X but present in both *D. pseudoobscura* and *D. affinis*, suggesting that it was deleted on the neo-X after the *D. pseudoobscura -D. miranda* split. The deletion is 2.4 kb in size and includes all of FBpp0276436 on the neo-X. In males, two transcripts were expressed that match FBpp0276435/FBpp0276436. The first, shorter transcript (355bp), is 1.7% diverged from the region in *D. pseudoobscura* that is deleted on the neo-X, but it has no blat hit with the *D. miranda* assembled genome sequence; we conclude that this must be a neo-Y copy homologous to FBpp0276436. The other, longer transcript recovered in *D. miranda* males (1,200 bp long), is 7.3% diverged from the *D. miranda* genomic neo-X sequence, 7.2% from *D. pseudoobscura* FBpp0276435 and 5.6% from *D. pseudoobscura* FBpp0276436. It is probably a transcript chimeric of FBpp0276435/ FBpp0276436 on the neo-Y because our method to assemble the neo-Y transcripts is not able to distinguish between true neo-X-neo-Y divergence and neo-Y-neo-Y paralog divergence for cases in which one of the neo-X paralogs has been deleted, as here.
